# No difference in the intention to engage others in academic transgression among medical students from neighboring countries: a cross-national study on medical students from Bosnia and Herzegovina, Croatia, and Macedonia

**DOI:** 10.3325/cmj.2016.57.381

**Published:** 2016-08

**Authors:** Varja Đogaš, Doncho M. Donev, Sunčana Kukolja-Taradi, Zoran Đogaš, Vesna Ilakovac, Anita Novak, Ana Jerončić

**Affiliations:** 1 Department of Psychological Medicine, University of Split School of Medicine, Split, Croatia; 2 Institute of Social Medicine, Faculty of Medicine, Skopje, Macedonia; 3 Department of Physiology and Immunology, University of Zagreb Medical School, Zagreb, Croatia; 4 Department of Neuroscience, University of Split School of Medicine, Split, Croatia; 5 Department of Medical Statistics and Medical Informatics, University of Osijek School of Medicine, Osijek, Croatia; 6 Department of Clinical Microbiology, University Hospital Centre Split, Split, Croatia; 7 Department of Microbiology, University of Split School of Medicine, Split, Croatia; 8 Department of Research in Biomedicine and Health, University of Split School of Medicine, Split, Croatia

## Abstract

**Aim:**

To asses if the level of intention to engage others in academic transgressions was comparable among medical students from five schools from neighboring Southern-European countries: Croatia, Bosnia and Herzegovina, and Macedonia; and medical students from western EU studying at Split, Croatia.

**Methods:**

Five medical schools were surveyed in 2011, with ≥87% of the targeted population sampled and a response rate of ≥76%. Students’ intention to engage a family member, friend, colleague, or a stranger in academic transgression was measured using a previously validated the Intention to Engage Others in Academic Transgression (IEOAT) questionnaire and compared with their intention to ask others for a non-academic, material favor. Data on students’ motivation measured by Work Preference Inventory scale, and general data were also collected. Multiple linear regression models of the intention to engage others in a particular behavior were developed.

**Results:**

The most important determinants of the intention to engage others in academic transgression were psychological factors, such as intention to ask others for a material favor, or students’ motivation (median determinant’s β of 0.18, *P* ≤ 0.045 for all), whereas social and cultural factors associated with the country of origin were either weak (median β of 0.07, *P* ≤ 0.031) or not relevant. A significant proportion of students were aware of the ethical violations in academic transgressions (*P* ≤ 0.004 for all transgressions), but a large proportion of students also perceived academic cheating as a collective effort and were likely to engage people randomly (*P* ≤ 0.001 for all, but the most severe transgression). This collective effort was more pronounced for academic than non-academic behavior.

**Conclusion:**

Culture differences among neighboring Southern-European countries were not an important determinant of the intention to engage others in academic cheating.

Academic dishonesty and misconduct is a growing and widespread phenomenon in medical and health care schools worldwide (1-4). Up to 58% of medical students in the USA self-reported some form of academic misconduct, with up to 88% admitting to cheating at least once in college (5,6). Other studies also found a high prevalence of self-reported misconduct at medical schools, ranging from 20% in Ethiopia (7) to 97% in Croatia (3). While such large variation of prevalence between countries can, at least partly, be attributed to difference in questionnaires, there is no doubt that the overall prevalence of academic misconduct is worrisomely high. This is even more the case as it has been shown that cheating at medical school predicted disciplinary actions by national medical boards since some of the students who used to cheat followed the same behavior pattern in their professional carriers (8).

Currently, the Theory of Planned Behavior (TPB) developed by Ajzen (9) is the most promising theoretical framework explaining academic misconduct of university students. According to the TPB, a person’s intention to perform a specific misconduct behavior precedes actual engagement in the behavior. The intention is perceived by the Theory as the central latent construct, which is shaped by the person’s attitudes toward the specific behavior, perception of the social norms governing the behavior, perceived moral obligation to perform/not perform specific behavior, and the perceived degree of control over the behavior. Although theoretical models could provide a rationale for misbehavior, their predictive power, however, is still not at the desired level. Due to lack of studies using the TPB model to explain cheating behaviors of medical students, studies investigating this phenomenon in medical students are exploratory in nature.

Predictors of academic misconduct in students identified in various exploratory studies include grades, motivation, personality types, and gender, as well as contextual factors, such as the risk of detention, peer behavior, socioeconomic environment, or educational system (10-26). Studies on general ethical decision-making indicated culture as one of the most important variables influencing misconduct processes (27,28), with society’s cultural dimension Individualism vs Collectivism (IDV) being the most important factor (29). A comparative study on academic misconduct at Lebanon and US universities found that cultural differences were strong determinants of overall prevalence of misconduct and students’ attitudes toward this behavior (30). These were the differences in individual or social value systems, and orientation of society toward individualism or collectivism. Likewise, the largest cross-cultural study performed on 7213 business students from 21 countries revealed significant cross-country heterogeneity in the average prevalence of copying, with the probability of cheating ranging from 88% at Eastern European universities (Poland, Romania, and Slovenia) to 5% at Scandinavian universities (26). The strong effect of culture on academic misconduct was recently corroborated by several studies, with students from “Individualist” western societies (societies scoring above 50 on IDV scale) usually reporting more negative attitudes toward cheating than students from “Collectivist” societies (31-37).

Regardless of the potential of cross-cultural studies to investigate the cultural determinant of academic transgression and to yield less biased results due to application of uniform analysis techniques on different populations, such studies are scarce, primarily performed on business students, and oriented toward comparison of attitudes between the US and some other country(ies) rather than toward the actual behavior. No cross-cultural study has so far investigated misconduct in medical students.

The aim of this study was to compare the intention to engage others in academic misconduct among medical students from neighboring post-communist countries in Southeastern Europe: Croatia, Bosnia and Herzegovina (BH), and Macedonia; and a control group of medical students from western EU. We hypothesized that the level of intention to perform such behavior was comparable among tested students.

## Methods

### Participants

A cross-sectional study was performed during 2011 at three medical schools in Croatia (Universities of Zagreb, Split, and Osijek) and one medical school in both Bosnia and Herzegovina (University of Mostar) and Macedonia (University of Skopje). The target population included medical students attending the first, the third, or the last, the sixth study year at these medical schools. We also surveyed medical students attending the first year of the international medical study program at the University of Split, Medical School, which represented a control group predominantly from western EU countries and with various cultural identities. A convenience, non-random sample of the targeted student groups was collected, with students surveyed at obligatory lectures/seminars when the highest attendance was expected. Students were asked to voluntarily complete an anonymous, self-administered questionnaire. The same approach and introductory lines by investigators were used for all tested groups. At all schools ≥87% of the targeted population was sampled. The study was approved by the Ethics Committee of the University of Split, School of Medicine.

### The primary outcome

The primary outcome of the study was the level of intention to engage others in a behavior. Intention is a central construct in TPB models of human behavior and its reporting is expected to be less confounded than self-reporting of the actual behavior. We compared the level of intention to engage others into academic transgression with the level of intention to ask for a personal material favor as an example of intention that was non-related to academic cheating. The level of intention to engage others into academic transgression was also correlated with individual factors indicated as potential predictors of student cheating: gender, study year, academic success, and motivation for study/work.

### Questionnaire

We used a questionnaire described in detail in our previous study (38), which consisted of three parts: a) general data, b) questionnaire about students' intentions to perform a particular behavior by engaging others – Intention to Engage Others in Academic Transgression (IEOAT) (38), and c) validated questionnaire on motivational orientation – Work Preference Inventory (WPI) (39).

General data included age, gender, study year, and grade point average (GPA) at the study year immediately preceding the tested one that was obtained either at the medical school or, for the first year students, in secondary school.

IEOAT questionnaire tests students' intentions to perform a particular behavior by engaging four types of individuals: 1. family member or close relative, 2. close friend, 3. colleague, and 4. stranger. Two groups of tested behaviors were: a) four academic transgressions, and b) two behaviors that were not related to academic misconduct, but involved asking others for a personal material favor. The questionnaire used dichotomous YES/NO questions for each scenario and was piloted with 4 educators and students from the University of Split Medical School to improve the clarity, consistency, and face validity of the questionnaire. Its validity was tested in our previous study (38) and again confirmed in this study.

Four academic transgressions (A1-A4) were chosen from each of the four specific clusters from the hierarchical cluster analysis of 11 self-reported cheating behaviors at the University of Zagreb School of Medicine (40): A1. Would you ask others to sign the lecture attendance sheet instead of you in your absence?, A2. Would you ask others to let you copy answers during a text exam?, A3. Would you ask others to use the cells phone to send answers to you during a test?, A4. Would you ask others to use personal connections with the examiner to arrange you to pass the exam? The favors were ordered from the least (A1) to the most (A4) serious transgressions. The scale ranged from 0 (would not ask anyone) to 16 (would ask all four types of persons in all four situations). The maximum score corresponded to answering YES in all four cheating scenarios for all four types of persons to be engaged in the cheating behavior.

Everyday personal material favors presented to the respondents were: P1. Would you ask others to lend you €50 for three days?, P2. Would you ask others to lend you a car for a day? The scale ranged from 0 (would not ask anyone) to 8 (would ask all four types of persons in both scenarios).

The third part of the questionnaire was the WPI for assessing individual differences in college students’ intrinsic and extrinsic motivational orientations (39). It consists of 30 statements, in which the examinee is asked to choose in which extent a statement describes her or him: 1. never or almost never; 2. sometimes; 3. often; and 4. always or almost always, and the score for each statement ranges from 1 (completely disagree) to 4 (completely agree). The questionnaire was scored on two primary scales (intrinsic and extrinsic motivational orientation, each with 15 statements), each subdivided into 2 subscales: outward and compensation for extrinsic motivation and enjoyment and challenge for intrinsic motivation (10 and 5 statements, respectively).

### Data analysis

Cronbach α was used to estimate the internal consistency of the academic and the non-academic scale on students' intentions. The χ^2^ test was used to evaluate the differences in frequencies of medical students by country, or by other categorical variables (ie, while estimating gender distribution within the country). After checking if the skewness of an analyzed quantitative variable divided its their standard error was less than the threshold of 2.5 (as recommended to detect significant deviation from normality in large samples) (41), we proceeded with parametric testing. Specifically, differences in means of score variables were tested either by *t* test (2 groups) or ANOVA (>2 groups) followed by *post-hoc* testing.

Average per-item scores were generated by summing the answers on a particular scale and dividing them by the total number of items. For both intention scales (academic transgression or personal material favors), overall per-item scores ranged from 0 to 1 and represented the proportion of positive answers. For motivational (sub)scales, averaged per-item scores ranged from 1 (never or almost never) to 4 (always or almost always). Per-item scores were introduced in order to compare the intensities between different (sub)scales.

To evaluate potential factors influencing the intention of engaging others in academic transgression we performed a stepwise multiple linear regression (MLR) model of the summed score of academic transgression intentions, with intentions to ask for personal material favors, gender, study year, repetition of the year, GPA, and motivational (sub)scores as independent variables. The same statistical method was applied to the summed score of intentions to ask for a personal material favor. The score data were first logarithmically transformed to correct for non-normality of error distribution and the following variables were used as independent variables: gender, study year, repetition of the year, GPA, and motivational (sub)scores. All the assumptions regarding the appropriate usage of ANOVA and MLR methods including the normality assumptions were carefully checked as described previously. Statistical analyses were performed using SPSS version 19.0 (IBM, Armonk, NY, USA). The level of significance was set at *P* = 0.050.

## Results

### Respondents

1545 questionnaires were collected. 38 questionnaires were excluded from the analysis due to abundance of missing answers, which resulted in 1507 valid questionnaires. Country-specific response rates were ≥76%.

Most of the respondents were women (Table 1, χ^2^ test, *P* ≤ 0.002), in accordance with the typical gender structure of medical students in the region (40). Most of the students had high GPA, which translates to the grade “very good” in the majority of educational systems, and had a high average score on the overall motivation scale ranging from 59% to 67% of the maximum score.

**Table 1:** The characteristics of study participants by the country of origin

Characteristic	Western European Union origin, n = 60	Croatia, n = 829	Bosnia and Herzegovina, n = 121	Macedonia, n = 497
N (%) or mean ± standard deviation				
Gender				
men	29 (48)	266 (32)	41 (34)	177 (36)
women	29 (48)	553 (67)	80 (66)	316 (64)
missing data	2 (4)	10 (1)	0 (0)	4 (1)
Age, years	20.6 ± 2.1	21.1 ± 2.5	21.3 ± 2.4	21.7 ± 2.9
Study year				
1st	59 (100)	295 (36)	47 (39)	159 (32)
3rd	-^†^	298 (36)	44 (36)	161 (32)
6th	-	235 (28)	30 (25)	177 (36)
Repetition of the year				
no	54 (93)	733 (88)	86 (71)	401 (81)
yes	4 (7)	81 (10)	33 (27)	90 (18)
missing data	0 (0)	15 (2)	2 (1)	6 (1)
Grade point average (scale from 1 to 5), mean±SD	NA*	4.2 ± 0.6	3.8 ± 0.7	3.9 ± 0.9^‡^
Score on academic cheating (scale from 0 to 16)	6.3 ± 3.8	6.7 ± 3.5	6.2 ± 3.7	6.3 ± 3.8
Score on personal favors (scale from 0 to 8)	4.3 ± 1.2	2.9 ± 1.6	2.8 ± 1.7	2.3 ± 1.8
Score on overall motivation (scale from 30 to 120)	83.0 ± 7.4	85.7 ± 8.3	85.4 ± 8.2	89.6 ± 7.9

*NA – not applicable due to differences in grading scales from various educational systems.

†Since the international study program just started there were no students enrolled at higher study years.

‡Grades are transformed to the grading scale used in Croatia, ranging from 1 (fail) to 5 (excellent).

### Asking for academic and personal favors

Internal consistency of the academic cheating survey was high. The overall Cronbach α for 16 items in the academic cheating survey was 0.845 (95% confidence interval [CI] 0.833-0.856). For 8 items in the personal favors survey, the overall Cronbach α was 0.723 (95% CI 0.701-0.743), also indicating a satisfactory level of consistency. All items had item-total correlations of r_pb_≥0.30 and no item had α-if-item-deleted greater than the overall α.

Respondents’ mean ± standard deviation (SD) score of academic cheating on a scale from 0 to 16 was 6.5 ± 3.6 (achieving on average 41% of the maximum score), whereas the score on personal favors on a scale from 0 to 8 was 2.8 ± 1.7 (achieving 35% of the maximum score). When translated to per-item score, intensities of intentions were 0.40 ± 0.23 for academic transgressions and 0.35 ± 0.21 for non-academic personal material favors. Hence, the overall intention to perform academic transgression was significantly higher than the intention to ask for a material favor (*t* test, *P* < 0.001).

**Figure 1:**
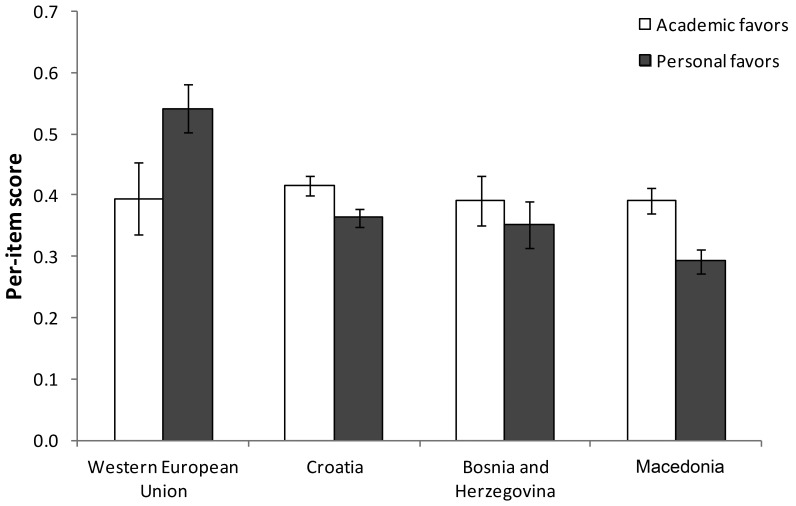
Mean per-item scores (scale from 0 to 1) with accompanying 95% confidence intervals (CI) for intention to engage others in academic cheating (white bars) or ask for personal favors (gray bars) – shown by the country of origin. Countries are ordered by geographic location – from west to the east.

With regard to the country of origin, we found no difference in scores on intention to engage others in academic cheating (ANOVA, *P* = 0.242; Figure 1). However, we found a gradual decline in mean per-item score on intention to ask others for a personal material favor from the westernmost to the easternmost country of origin (Figure 1). This trend, depicted by the curvilinear decrease from the westernmost to easternmost geographic location, was significant (trend analysis, weighted linear *P* < 0.001 and quadratic term *P* = 0.002). Additionally, *post hoc* tests revealed that students from western EU countries had higher scores than students from other countries (*post hocP*≤6.8 × 10^−8^), whereas Macedonian students had significantly lower scores than students from other countries (*P* ≤ 0.029). No difference in scores was observed between Croatian and BH students (*P* = 0.996).

### The pattern of engaging other people

We found a similar pattern of responses across the countries regarding types of students who would engage others in a particular behavior (Table 2). In general, participants who would not engage anyone in an academic transgression were more prevalent than was expected by chance alone, indicating students’ awareness of the ethical violation in particular scenarios. In addition, a sharp distinction in responses for less and more serious transgressions was evident: only 14%-16% of students on average would not engage anyone in signing attendance sheet or copying answers, but 43%-59% of students would not engage anyone in sending answers by cell phone or using a personal connection to pass an exam. The discrepancy between less and more serious transgressions was also evident when we analyzed responses of students intending to engage one or more persons. For less serious transgressions, respondents made the following choices more frequently than expected by chance: they would engage one (a friend), two (a family member and a friend), three (a family member, a friend and a colleague), or four persons (anyone); whereas for the most serious transgression students would not engage more than two persons. In addition, while the most prevalent choice for less serious transgressions was the engagement of two persons (a family member and a friend), for more serious transgressions, the higher was the number of persons engaged, the lower was the prevalence of that particular choice.

**Table 2:** Responses that were more prevalent than expected by chance alone, shown by scenario and by country

		Macedonia, n = 497	Bosnia and Herzegovina, n = 121	Croatia, n = 829	West European Union, n = 60
**Scenario:**	**Would engage:**	**N (% of total responders by country)**			
**Academic transgressions**					
Sign a lecture attendance sheet	no one	**56 (11)^‡^**	**26 (21)**	39 (5)	**10 (17)**
	friend	**96 (19)**	10 (8)*	**71 (9)**	0 (0)
	family and friend	**127 (26)**	**46 (38)**	**287 (35)**	**19 (32)**
	family, friend and colleague	**93 (19)**	**19 (16)**	**298 (36)**	**13 (22)**
	anybody	**56 (11)**	**9 (7)**	**90 (11)**	5 (8)
Let copy answers during test exam	no one	**98 (20)**	**16 (13)**	**115 (14)**	**11 (18)**
	friend	**51 (10)**	**13 (11)**	58 (7)	3 (5)
	family and friend	**107 (22)**	**32 (26)**	**233 (28)**	**12 (20)**
	family, friend and colleague	**57 (11)**	**26 (21)**	**166 (20)**	**17 (28)**
	anybody	**120 (24)**	**27 (22)**	**221 (27)**	**9 (15)**
Send answers by cell phone during test exam	no one	**217 (44)**	**40 (33)**	**371 (45)**	**29 (48)**
	family and friend	**117 (24)**	**43 (36)**	**233 (28)**	**15 (25)**
	family, friend and colleague	**42 (8)**	11 (9)	**87 (10)**	**12 (20)**
	anybody	**47 (9)**	11 (9)	**84 (10)**	0 (0)
Use personal connection to pass exam	no one	**263 (53)**	**78 (64)**	**616 (74)**	**26 (43)**
	friend	**54 (11)**	9 (7)	49 (6)	**8 (13)**
	family and friend	**122 (25)**	**22 (18)**	**107 (13)**	**16 (27)**
**Personal favors**					
Lend car for a day	no one	**181 (36)**	**25 (21)**	**159 (19)**	1 (2)
	friend	**88 (18)**	**16 (13)**	**165 (20)**	5 (8)
	family and friend	**172 (35)**	**64 (53)**	**441 (53)**	**36 (60)**
	family, friend and colleague	19 (4)	3 (2)	17 (2)	**16 (27)**
Lend money (€) for three days	no one	**165 (33)**	**30 (25)**	**175 (21)**	0 (0)
	friend	23 (5)	**16 (13)**	**60 (7)**	1 (2)
	family	**78 (16)**	**19 (16)**	**118 (14)**	**8 (13)**
	family and friend	**194 (39)**	**48 (40)**	**427 (52)**	**37 (62)**
	family, friend and colleague	18 (4)	1 (1)	22 (3)	**12 (20)**

*Regular font style – responses that were more prevalent than expected by chance (6%) but did not reach statistical significance.

‡Bold font style – responses that were significantly (*P* < 0.05) more prevalent than expected by chance.

It is worth noting that students who did not differentiate between different types of relationships and would include all four types of individuals were in general significantly more prevalent than expected. Only for the most serious transgression – use of personal connections to pass an exam – the participants tended not to engage others randomly in a behavior.

With regard to asking others for a personal material favor, students were also aware that such behaviors were not commonly accepted, as a significantly higher percentage of students would not ask anybody for a favor (Table 2). The exceptions were international students from western EU countries who would, as a rule, ask someone to lend them money or a car. Also, students were more prone to engage only two types of close individuals – a close friend and a family member (on average 49% of all responses) when asking for a material favor. This was in contrast to less serious academic transgressions, in the case of which a significant portion of students from all countries did not differentiate between types of individuals and would include all four types. Again, the exception were international students from western EU countries who would engage from 2 to 4 individuals at a significantly higher rate than expected by chance.

### Descriptors of academic cheating and asking for personal favors

We demonstrated that the determinants of increased level of intention to engage others in academic cheating were higher propensity to ask for a personal favor and higher extrinsic motivation (Table 3). Contrary to this, higher intrinsic motivation was associated with a lower level of intention.The strongest determinant of academic cheating was the intention to ask for a personal favor, followed by extrinsic and intrinsic motivation, whereas country of origin, age of participants, GPA, gender, study year, or repetition of the study year were not identified as determinants. However, when we stratified the data by country, both the fitness of the stepwise model and its significant determinants differed between the countries (Table 3, country-specific models). Overall, psychological factors such as the intention to ask for a personal favor and motivation were present in all country-specific models (median determinants’ β of 0.18, *P* ≤ 0.045 for all), whereas significant sociological determinants (median β of 0.07, *P* ≤ 0.031) were either unique to a particular country model (eg, gender in Croatia) or quite differently affected the intention to engage others in different countries (eg, year of the study was a positive predictor in Croatia and negative in Macedonia).

**Table 3:** Determinants of respondents' (n = 1507) level of intention to engage others in academic cheating, or asking for a personal favor – ordered by determinant’s strength, from the strongest to the weakest

		Model fit, adjusted R^2 ^(%)	Determinants	B	Beta	*P*-value	95% confidence interval for B	
Academic cheating	Joined model	13	score on personal favors	0.62	0.29	<0.001	0.52	0.72
			score on total extrinsic motivation	0.11	0.16	<0.001	0.08	0.14
			score on total intrinsic motivation	-0.09	-0.13	<0.001	-0.12	-0.06
	Western European Union	NA*	NA					
	Croatia	14	score on personal favors	0.53	0.24	<0.001	0.39	0.67
			score on total extrinsic motivation	0.11	0.18	<0.001	0.07	0.15
			score on total intrinsic motivation	-0.10	-0.17	<0.001	-0.14	-0.06
			study year	0.32	0.07	0.028	0.04	0.60
			gender = male	0.53	0.07	0.031	0.05	1.01
	Bosnia and Herzegovina	9	score on personal favors	0.49	0.23	0.011	0.12	0.87
			repetition of the year = YES	1.85	0.23	0.011	0.43	3.27
	Macedonia	18	score on personal favors	0.77	0.36	<0.001	0.59	0.94
			repetition of the year = YES	-1.23	-0.12	0.005	-2.07	-0.38
			study year	-0.44	-0.09	0.029	-0.84	-0.05
			score on total extrinsic motivation	0.06	0.08	0.045	0.00	0.13
Personal favors	Joined model	7	western EU origin	1.35	0.15	<0.001	0.92	1.78
			Macedonian origin	-0.55	-0.15	<0.001	-0.73	-0.37
			gender = male	0.44	0.12	<0.001	0.27	0.62
			repetition of a year = YES	-0.27	-0.06	0.027	-0.5	-0.03

*NA – model was not applied due to small sample size.

With regard to the intention to ask for a personal favor, western EU origin was associated with higher, whereas Macedonian origin, female gender, and repetition of a year were associated with a lower level of intention (adjusted R^2^ = 7%) (Table 3). Motivational (sub)score, study year, or GPA were not significant predictors of the intention to ask for a personal favor.

### Respondents’ motivation for work/study

**Figure 2:**
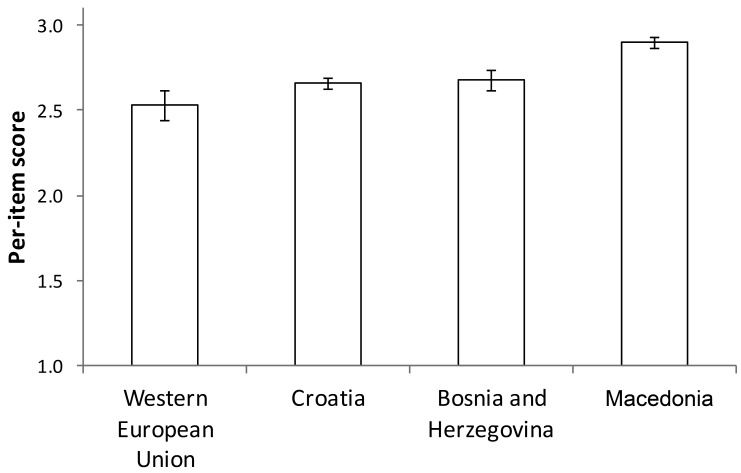
Mean per-item score (scale from 1 to 4) with accompanying 95%confidence intervals (CI) on the extrinsic outward motivational subscale – shown by the country of origin.

Except for per-item scores on intrinsic motivation challenge subscale (one-way ANOVA; *P* = 0.058) we found significant country-specific differences in per-item scores on the overall motivation scale or on its subscales (*P* ≤ 0.004). As a rule, per-item motivation (sub)scores were the highest in the Macedonian sample, lower in BH and Croatia, and the lowest in the students from western EU countries (Table 4). This cross-countries pattern was observed for the overall score, all extrinsic (sub)scales, and the intrinsic enjoyment subscale. In fact, trend analysis confirmed the significance of these trends and revealed that the most prominent west-east change was observed on the extrinsic outward subscale (Table 4, Figure 2). In all countries, the largest per-item motivation score was observed on the intrinsic enjoyment subscale, whereas the lowest was observed on the intrinsic challenge subscale.

**Table 4:** Mean per-item score on different motivational (sub)scales and accompanying 95% confidence intervals (CI), shown by country. Also shown are significant trends as determined by trend analysis

	Western European Union		Croatia		Bosnia and Herzegovina		Macedonia		Trend analysis – significant trends
	mean±SD*	95% CI	mean±SD	95% CI	mean±SD	95% CI	mean±SD	95% CI	
**Overall score**	2.77 ± 0.25	(2.70, 2.83)	2.86 ± 0.28	(2.84, 2.88)	2.85 ± 0.27	(2.80, 2.89)	2.99 ± 0.26	(2.96, 3.01)	linear *P* < 0.001
**Overall extrinsic**	2.55 ± 0.35	(2.46, 2.64)	2.67 ± 0.38	(2.65, 2.70)	2.70 ± 0.34	(2.64, 2.76)	2.88 ± 0.33	(2.85, 2.91)	linear *P* < 0.001, quadratic *P* = 0.045
**Extrinsic outward**	2.53 ± 0.38	(2.44, 2.63)	2.66 ± 0.41	(2.63, 2.69)	2.68 ± 0.36	(2.62, 2.74)	2.90 ± 0.37	(2.87, 2.93)	linear *P* < 0.001, quadratic *P* = 0.023
**Extrinsic compensation**	2.59 ± 0.53	(2.46, 2.73)	2.71 ± 0.53	(2.67, 2.75)	2.73 ± 0.50	(2.64, 2.82)	2.83 ± 0.49	(2.79, 2.88)	linear *P* < 0.001
**Overall intrinsic**	2.98 ± 0.31	(2.90, 3.06)	3.04 ± 0.38	(3.01, 3.07)	2.99 ± 0.38	(2.93, 3.06)	3.10 ± 0.33	(3.07, 3.12)	linear *P* = 0.003
**Intrinsic enjoyment**	3.17 ± 0.30	(3.09, 3.24)	3.27 ± 0.41	(3.24, 3.30)	3.27 ± 0.41	(3.19, 3.34)	3.36 ± 0.38	(3.32, 3.39)	linear *P* < 0.001
**Intrinsic challenge**	2.60 ± 0.51	(2.47, 2.73)	2.58 ± 0.51	(2.54, 2.61)	2.45 ± 0.58	(2.34, 2.55)	2.57 ± 0.51	(2.53, 2.62)	

*SD – standard deviation.

## Discussion

This study found no difference in the intention to engage others in academic misconduct among students from Croatia, BH, Macedonia, or those of western-EU origin. The main determinant of this intention was a higher score on the intention to ask for a personal material favor (accounting for the half of the total variance in the regression model), followed by intrinsic and extrinsic motivation. Unlike these psychological determinants, GPA, country of origin, gender, or study year were not identified as relevant in the overall model. Stratified analysis by country, however, showed that, along with psychological determinants that were constantly strongest in all country-specific models (accounting for 83%-100% of total variance in country-specific models), collective factors such as study year or gender were also weak determinants. Moreover, it was evident that collective factors interacted quite differently with different cultures as they were either determinants of cheating unique to a particular country or conversely affected the intention to cheat in different countries. Thus, cultural differences between neighboring countries were not *per se* the determinants of the intention to engage others in cheating. Instead, it was the diverse and complex interaction of social and cultural factors that weakly and quite differently affected this intention and resulted in collective factors being irrelevant in a large cross-national model.

We observed similar results in our previous study in which the same regression model was built upon 2008 data of medical students from Croatia (University of Zagreb School of Medicine) who were surveyed with the same questionnaires (IEOAT and WPI). Specifically, the strength and significance of psychological determinants and gender were quite similar to our current model, whereas the results for study year and GPA were inconsistent (38). Therefore, not only that the psychological factors were the constant and strongest determinants of cheating in the same country, but the collective factors that were sensitive to different cultural settings were probably also affected by time changes too.

The majority of studies that observed large differences in attitudes and/or cheating behavior compared students from culturally very different countries, such as Western and Eastern countries. Teixeira et al (26) have recently performed a comprehensive study on economics and business students from 21 countries and identified striking differences in cheating behaviors between blocks of neighboring countries, ie, between Scandinavian and Eastern European countries, with countries within a block exhibiting quite similar prevalence of cheating, which is in line with our findings. A self-reported incidence of cheating and intention to help with cheating were also comparable among economics and business students from neighboring transitional economies: Belarus, Latvia, Lithuania, Russia, and Ukraine (37).

These similarities in intention to cheat among neighboring countries can be explained by the rationale of TPB (9). Continuing the work of Hofstede et al (29), who found that the most important feature by which a culture influences behavior is a society’s cultural dimension IDV, TPB considers that social influence, such as social norm and normative belief, is based on IDV-related variables. Differences in these variables are usually detected when quite different societies are compared. Therefore, it is not surprising for neighboring countries with a shared history until the war in 1991 that the differences in IDV-related variables are either nonexistent or not large enough to be detected and that the level of individualism/collectivism is probably comparable. The possibility that our study had low power to detect social and/or cultural differences can be excluded as we clearly detected trends by countries in some of the observed traits. There is also a possibility that the fine differences in collectivistic culture-related variables do exist and that the variable “intention of asking a personal material favor,” in the regression model grasped both the attitudes toward cheating and the collectivistic cultural dimension of a society. Specifically, students who would likely include random individuals in a transgression (ie, strangers) clearly exhibited greater tendencies for “collective behavior.” If this is the case, the potential of this variable to detect subtle differences in collectivistic cultural dimension along with attitudes toward a behavior should be investigated.

In general, students were aware of the ethical violations in each academic transgression as they were likely not to engage anybody in a transgression at a rate that was higher than expected by chance. At the same time, a significant portion of students did not discriminate between different types of relationships and would engage anybody in a transgression. This pattern of choices was observed for all transgressions, except the most serious one. Depending on the transgression, from 9% to 22% of students on average would randomly engage any person, including a stranger, in a transgression. This fact suggests that these students do not perceive a particular behavior as an ethical violation but more as a collaborative effort. On the other hand, asking for a material favor was reserved for close relationships, such as friends and family members. These findings are in line with the results of McCabe’s surveys (24), which have found that most college students in the USA see collaborating with others, even when it is forbidden, as a minor offense or no offense at all. A similar pattern was observed in our 2008 study, thus indicating the robustness of this phenomenon (38).

Interesting findings of this study were west-to-east trends detected in students’ intention to ask for a material favor and motivation scores, the external outward score in particular. Although the existence of trends points toward a west-to-east gradation of some social or cultural factors in the surveyed countries, it is beyond the scope of this study to elaborate on them. It should, however, be noted that previous research has suggested a relationship between social contexts (eg, economic growth) and motives within populations. High achievement motive, which is according to McClelland's motivational needs theory (42) based on intrinsic motivation, has been associated with subsequent economic growth, which in turn increases power motive, an analogue of extrinsic motivation (43,44). Indeed, Macedonia, which exhibited the highest level of motivation, also experienced the fastest economic growth (*http://www.tradingeconomics.com*/).

Limitations of this study are those of exploratory studies investigating academic cheating. We used a convenient non-random sample in a cross-sectional study design, which poses a risk of bias. Nevertheless, by sampling students at obligatory lectures/seminars we collected ≥87% of the targeted populations and ensured representativeness of the sample, with possible underrepresentation of only a small fraction of frequently sick students. Another limitation was that in BH and Macedonia we used a sample that was representative only for the surveyed medical school. Since research of sensitive issues, such as academic transgressions requires the agreement of researchers, academic staff, medical students, and medical school management, cross-national studies do not sample a representative sample of entire student population from a particular country, but instead use a sample representative of one university/school and generalize the findings to an entire country (26,31). Therefore, conclusions drawn from one university data represent a valuable source of information in this field.

In conclusion, this cross-national study on medical students from neighboring countries showed that psychological rather than collective factors are expected to be predominant determinants of intention to engage others in cheating. Cheating was perceived as a collective effort in a significant portion of students who were likely to engage people randomly in such behavior, although there were still students who were aware of the ethical violations in academic transgression. We believe that the number of academic transgressions can be reduced using the successful academic integrity programs and policies, which should be shared among neighboring countries and further improved.
